# A Path to Resume Aesthetic Care: Executive Summary of Project AesCert Guidance Supplement—Practical Considerations for Aesthetic Medicine Professionals Supporting Clinic Preparedness in Response to the SARS-CoV-2 Outbreak

**DOI:** 10.1089/fpsam.2020.0239

**Published:** 2020-05-15

**Authors:** Jeffrey S. Dover, Mary Lynn Moran, Jose F. Figueroa, Heather Furnas, Jatin M. Vyas, Lory D. Wiviott, Adolf W. Karchmer

**Affiliations:** ^1^ SkinCare Physicians, Chestnut Hill, Massachusetts, USA.; ^2^ Department of Dermatology, Yale University School of Medicine, New Haven, Connecticut, USA.; ^3^ Brown Medical School, Providence, Rhode Island, USA.; ^4^ Department of Otolaryngology, Vanderbilt University School of Medicine, Nashville, Tennessee, USA.; ^5^ Department of Health Policy and Management, Harvard T.H. Chan School of Public Health, Harvard University, Boston, Massachusetts, USA.; ^6^ Department of Medicine, Brigham and Women's Hospital, Boston, Massachusetts, USA.; ^7^ Department of Surgery, Division of Plastic and Reconstructive Surgery, Stanford University School of Medicine, Palo Alto, California, USA.; ^8^ Division of Infectious Disease, Massachusetts General Hospital, Boston, Massachusetts, USA.; ^9^ Department of Medicine, Harvard Medical School, Boston, Massachusetts, USA.; ^10^ Department of Medicine, California Pacific Medical Center, San Francisco, California, USA.; ^11^ Harvard Medical School, Boston, Massachusetts, USA.; ^12^ Division of Infectious Disease, Beth Israel Deaconess Medical Center, Boston, Massachusetts, USA.

## Executive Summary of Project AesCert™ Guidance Supplement

This Project AesCert Guidance Supplement (“Guidance Supplement”) was developed in partnership with a multidisciplinary panel of board-certified physician and doctoral experts in the fields of infectious disease, immunology, public-health policy, dermatology, facial plastic surgery, and plastic surgery. The Guidance Supplement is intended to provide aesthetic medicine physicians and their staff with a practical guide to safety considerations to support clinic preparedness for patients seeking nonsurgical aesthetic treatments and procedures following the return-to-work phase of the coronavirus disease 2019 (COVID-19) pandemic, once such activity is permitted by applicable law. Many federal, state, and local governmental authorities, public-health agencies, and professional medical societies have promulgated COVID-19 orders and advisories applicable to health-care practitioners. The Guidance Supplement is meant to provide aesthetic physicians and their staff with an additional set of practical considerations for delivering aesthetic care safely and generally conducting business responsibly in the new world of COVID-19. The Guidance Supplement is published as an Appendix to this article and can be read in full online at www.liebertpub.com/fpsam

Aesthetic providers will face new and unique challenges as government stay-at-home orders and related commercial limitations are eased, the U.S. economy reopens, and health-care systems transition from providing only urgent and other essential treatment to resuming routine care and elective procedures and services. Medical aesthetic specialties will therefore wish to resume practice in order to ensure high-quality expert care is available and, importantly, to help promote patients' positive self-image and sense of well-being following a lengthy and stressful period of quarantine. In a number of areas, this Guidance Supplement exceeds traditional aesthetic office safety precautions, recognizing reduced tolerance in an elective treatment environment for any risk associated with COVID-19's highly variable presentation and unpredictable course. The disease has placed a disturbing number of young, otherwise healthy patients in extremis with severe respiratory and renal failure, stroke, pericarditis, neurologic deficits, and other suddenly life-threatening complications, in addition to its pernicious effects on those with pre-existing morbidities and advanced age. Accordingly, the Guidance Supplement seeks to establish an elevated safety profile for providing patient care while reducing, to the greatest extent reasonably possible, the risk of infectious processes to both patients and providers.

While the Guidance Supplement cannot foreclose the risk of infection or serve to establish or modify any standards of care, it does offer actionable risk-mitigation considerations for general office comportment and for certain nonsurgical procedures typically performed in aesthetic medical settings. It is axiomatic that all such considerations are necessarily subject to the ultimate judgment of each individual health-care professional based on patient situation, procedure details, office environment, staffing constraints, equipment and testing availability, and local legal status and public-health conditions.

Federal, state, and local government legal pronouncements and public-health conditions will inform the gating decisions of when permissible and prudent to reopen practices and re-engage with patients, and whether to limit certain procedures that may present greater contagion risk. While such gating decisions are not the focus of this Guidance Supplement, it is advisable that practices should consider, at a minimum, whether in their local communities: (1) new COVID-19 cases are declining sequentially to eliminate or at least substantially control community spread; (2) testing is available at a meaningful scale to validate perceived prevalence reductions; and (3) adequate protocols and resources are in place in conjunction with local health departments to conduct effective contact tracing where necessary in response to COVID-19 incidents. Without robust testing, the ability to identify individuals with COVID-19, do appropriate contact tracing, and isolate and treat the infected is substantially reduced. Therefore, in the absence of these enumerated local conditions, practices must factor cautiously the attendant increased risk of transmission into their reopening calculus.

Significantly, the principal variables within the control of the practicing aesthetic medicine physician are office and staff preparation, and communication and transparency with patients. The Guidance Supplement is focused heavily on these subjects, offering consensus guidance from authors representing relevant scientific and clinical disciplines.

The Project AesCert Guidance Supplement provides specific recommendations and considerations for preparing to reopen a medical aesthetic office and begin to deliver aesthetic patient care in a COVID-19 environment, including:

- Patient communication—establishing appropriate expectations for office visits and attendant risks;
- Clinic schedule management—considerations for schedule modification to convert non-treatment interactions to telehealth consultations, separate patients from one another in the office and avoid unnecessary staff contact;
- Facility management—physical modification of office common areas and treatment rooms, as well as check-in and check-out procedures, to promote safe practices and physical distancing;
- Cleaning procedures—discussion of disinfection methods and practices in each office area, ranging from medical instruments and treatment rooms to administrative items and reception areas;
- Personal Protective Equipment (PPE) for providers, staff and patients—recommendations for PPE types and use depending upon procedure-based risk assessment, and recognizing current global equipment shortages;
- Employee health screening and training—procedures and methods for identifying staff members who may be unwell before, during, and after work, and training of staff to identify potential COVID-19 presentation in coworkers, patients, and other office visitors; risks associated with exposure to known or suspected COVID-19-positive individuals are also discussed;
- Patient health and screening—procedures and methods for symptom recognition in patients before, during, and after office visits, with follow-up monitoring where appropriate;
- Remedial measures following onsite symptom presentation—a framework for addressing isolation of symptomatic individuals, office containment and disinfection, and contact tracing;
- Treatment room setup—preparing and securing treatment rooms for patient entry to contain office contamination and reduce overall potential COVID-19 exposure; and
- Aesthetic treatment considerations—pretreatment preparation and precautions, and other suggestions for minimizing risk of transmission in performing the most common types of office-based aesthetic procedures, such as neurotoxin and dermal filler injections, noninvasive body contouring, lasers and other similar energy-emitting devices, and a range of medical skin care treatments.

The Project AesCert Guidance Supplement also contains summary charts and checklists designed in collaboration by both infectious disease and aesthetic experts, which can be utilized immediately to assist office staff in understanding and modeling sound safety practices.

## Conclusion

Aesthetic medicine practices must navigate a daunting series of medical and business challenges occasioned by the COVID-19 pandemic. Most offices have been closed by operation of both common sense and legal requirement, as the public health community labors to comprehend both the magnitude and complexity of severe acute respiratory syndrome coronavirus 2 (SARS-CoV-2) and its sequelae. This crisis has created significant safety concerns and occasioned severe financial hardship for aesthetic physicians, staff, and patients alike. However, the authors posit that application of sound safety measures identified and considered in the Guidance Supplement will serve to assist aesthetic medicine specialties in returning to the delivery of patient care with reasonable risk-minimization strategies. It is critical that all disciplines of medicine, aesthetic and otherwise, share available information and work together to evolve effective approaches to practicing in a dramatically changed environment.

## Figures and Tables

**Figure:**
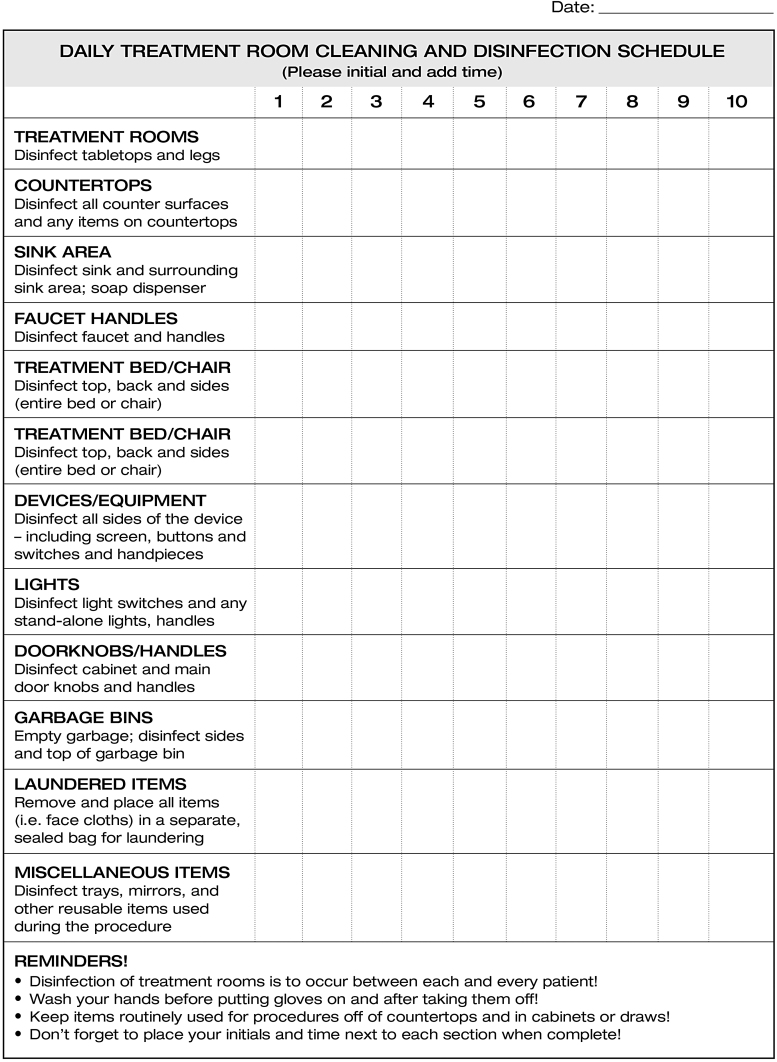
**Appendix Fig. A1.** Daily Treatment Room Disinfectant Checklist.

**Figure:**
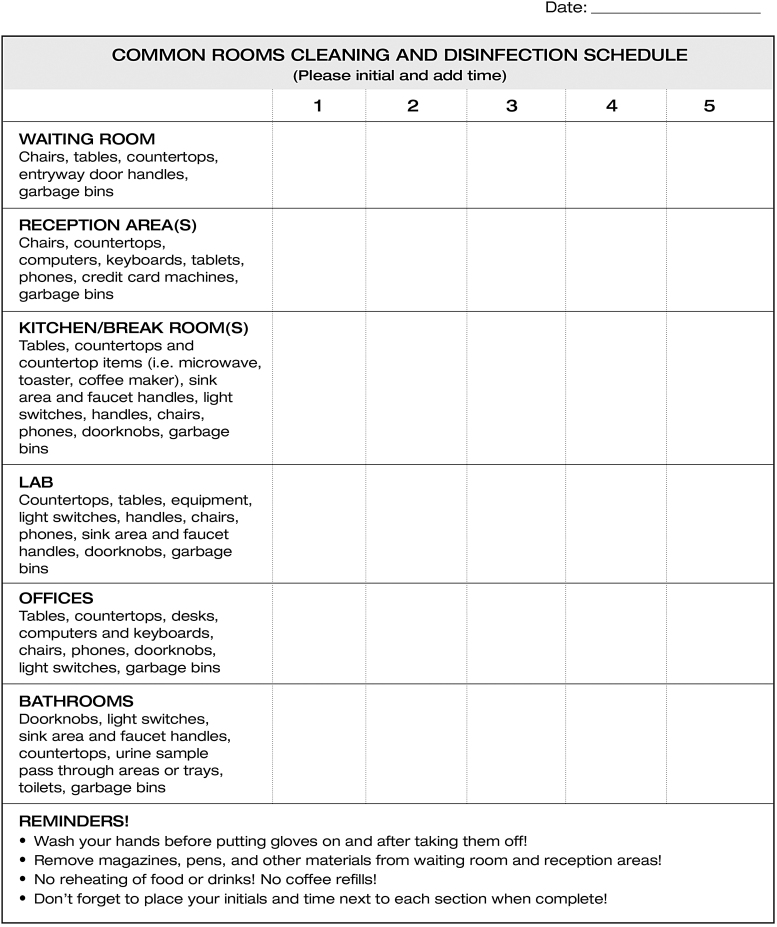
**Appendix Fig. A2.** Common Area Disinfectant Checklist.

**Figure:**
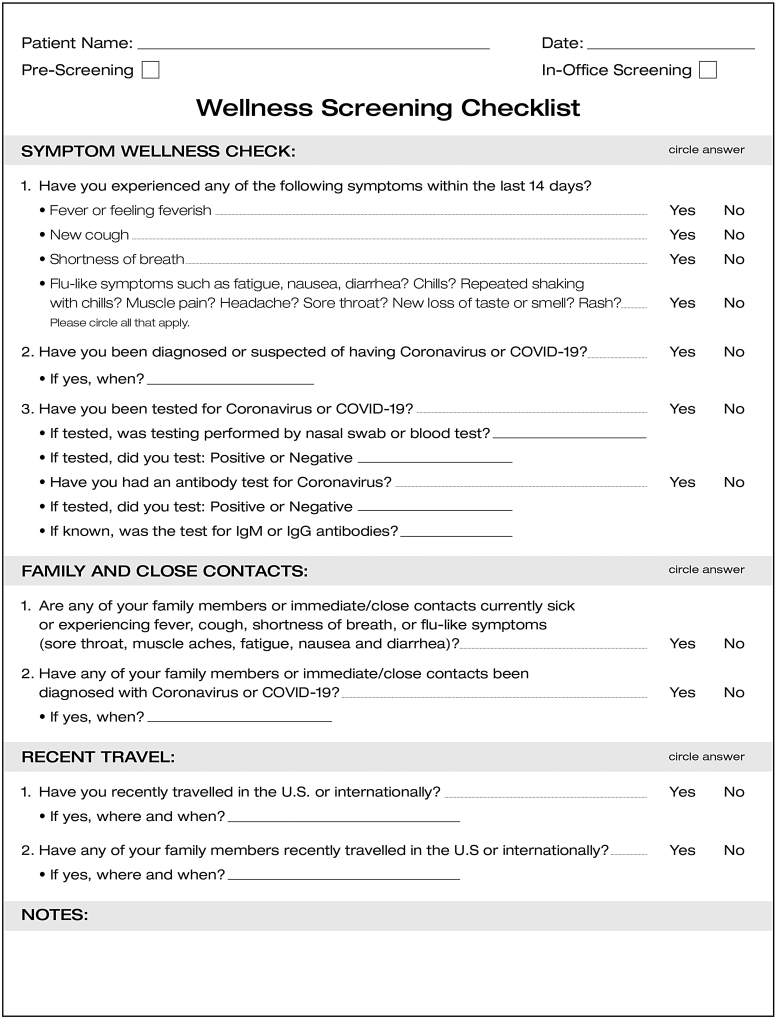
**Appendix Fig. A3.** Wellness Screening Checklist.

**Figure:**
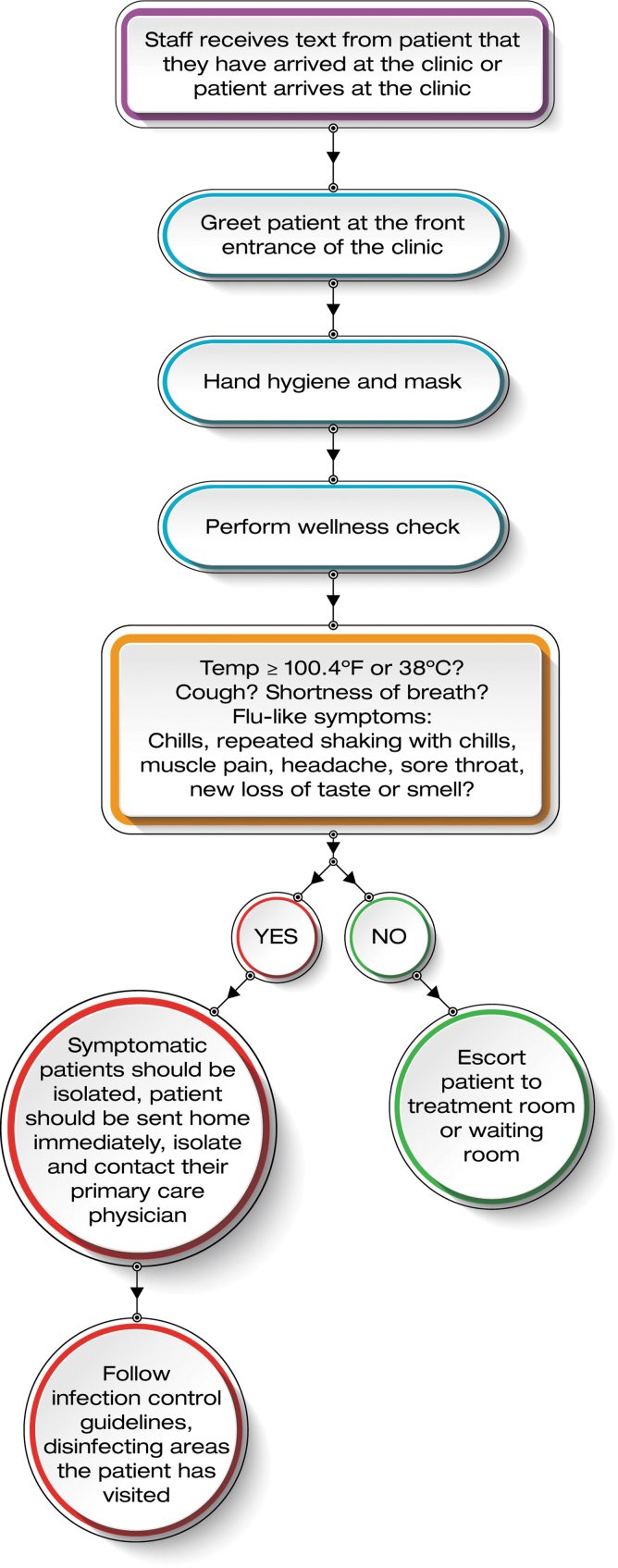
**Appendix Fig. A4.** Patient Screening Flow Chart.

**Figure:**
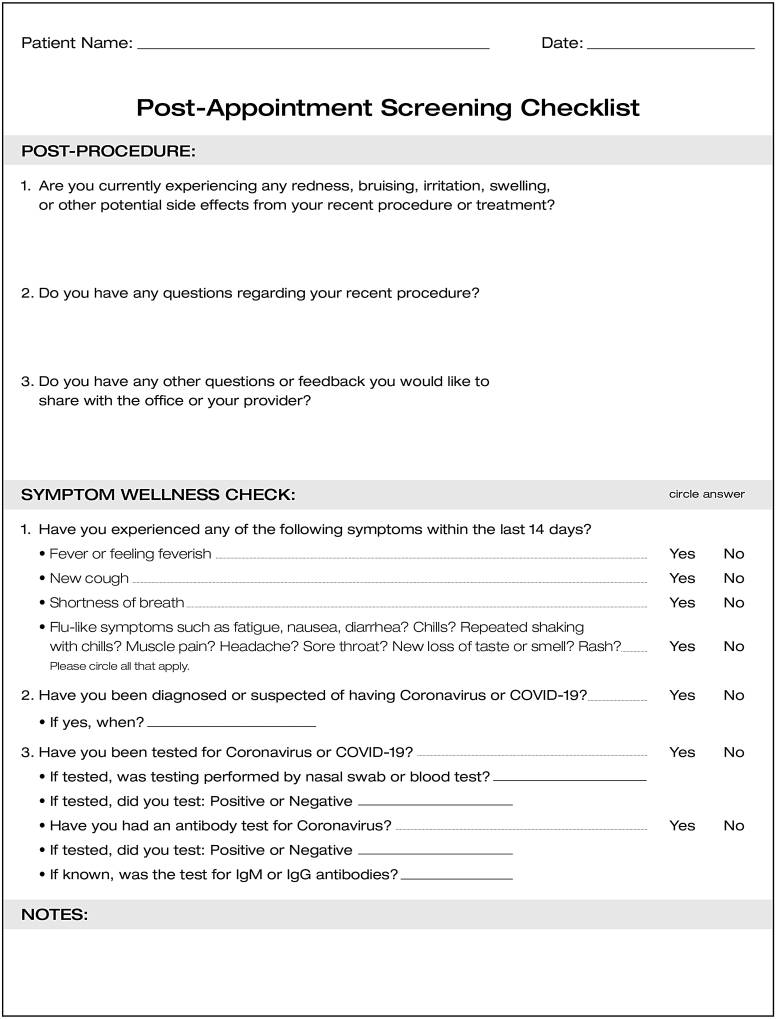
**Appendix Fig. A5.** Post-Appointment Screening.

## Appendix

### A PATH TO RESUME AESTHETIC CARE: Project AesCert Guidance—Practical Considerations for Aesthetic Medicine Professionals Supporting Clinic Preparedness in Response to the SARS-CoV-2 Outbreak

Copyright © 2020 The Skinbetter Science Institute™. All rights reserved.

#### Table of Contents

I. Statement of Purpose ............................................................ 128
II. Introduction ............................................................ 128
III. Medical Office Preparedness ............................................................ 129
Patient Communication and Transparency ............................................................ 129
 Managing the Office Environment - General Guidance ............................................................ 130
*Capsule Summary: Office Environment Considerations* ............................................................ 131
 Cleaning & Disinfecting Practices ............................................................ 132
 Personal Protective Equipment and Medical Supplies ............................................................ 133
*Capsule Summary: PPE and Medical Supply Considerations* ............................................................ 135
IV. Employee and Patient Health ............................................................ 135
 Employee Health and Training ............................................................ 135
 Recognition of Symptoms ............................................................ 136
 Symptomatic Patients and Employees ............................................................ 137
*Capsule Summary: Employee and Patient Health Considerations* ............................................................ 138
V. Patient Management Procedures ............................................................ 138
 Pre-Screening Patients ............................................................ 138
 Office Arrival, Check-In and Check-Out ............................................................ 138
*Capsule Summary: Patient Management Considerations* ............................................................ 139
VI. Clinical and Non-Surgical Treatments ............................................................ 140
 Treatment Room Set-Up ............................................................ 140
 Anesthesia and Analgesia ............................................................ 140
 Injectables ............................................................ 141
 Non-Invasive Body Contouring ............................................................ 141
 Energy-Based Procedures of the Face and Neck ............................................................ 142
 Skin Treatment Procedures ............................................................ 143
*Capsule Summary: Non-Surgical Treatment Considerations* ............................................................ 143
VII. Conclusions ............................................................ 145
VIII. References ............................................................ 145
IX. Appendix Figures ............................................................ 147
 Figure 1: Daily Treatment Room Disinfectant Checklist ............................................................ 147
 Figure 2: Common Area Disinfectant Checklist ............................................................ 148
 Figure 3: Wellness Screening Checklist ............................................................ 149
 Figure 4: Patient Screening Flowchart ............................................................ 150
 Figure 5: Post-Appointment Screening ............................................................ 151

### I. Statement of Purpose

This Project AesCert Guidance manuscript (“Guidance”) was developed in partnership with a multi-disciplinary panel of board-certified physician and doctoral experts in the fields of Infectious Disease, Immunology, Public Health Policy, Dermatology, Plastic Surgery and Facial Plastic Surgery. This Guidance is intended to provide aesthetic medicine physicians and their staffs with a practical guide to safety considerations to support clinic preparedness for patients seeking non-surgical aesthetic treatments and procedures following the return-to-work phase of the COVID-19 pandemic arising out of the novel coronavirus SARS-CoV-2, once such activity is permitted by applicable law.

Many federal, state and local governmental authorities, public health agencies and professional medical societies have promulgated COVID-19 orders and advisories applicable to health care practitioners, largely focused on the threshold determination of whether and when to reopen for business. These standards should be seriously considered, and where required by law or otherwise applicable or prudent, followed thoughtfully. This Guidance is not intended to contravene any such other mandates, which supersede this Guidance in the event of any conflict, but rather, to provide aesthetic physicians and their staffs with an additional set of practical considerations for delivering aesthetics care safely and generally conducting business responsibly in the new world of COVID-19.

### II. Introduction

Aesthetic physicians and their staff will face new and unique challenges as government stay-at-home orders and related commercial limitations are eased, and the U.S. economy reopens and healthcare systems transition from providing only urgent and other essential care to resuming routine care, elective procedures and services. Debate will continue about the wisdom, pace and scope of such reopening, but in the meantime patient demand for aesthetic treatments will return. The medical aesthetics specialties will therefore wish to resume practice in order to ensure high quality, expert care is available, and importantly to help promote patients' positive self-image and sense of well-being following a lengthy and stressful period of quarantine. In reopening aesthetic practices during the ongoing pendency of the COVID-19 outbreak, delivery of care must be accompanied by necessary precautions to safeguard the health and welfare of not only the patients and providers within the context of the office environment, but also the community at large with whom they interact immediately beyond the office walls.

There is widespread perception that, while aesthetic procedures are self-esteem and self-image enhancing, they are generally considered elective, with notable exceptions that may be deemed medically necessary (e.g., cases of congenital anomaly or traumatic injury). Because of their elective nature, extraordinary care must be taken to protect patients and healthcare professionals from COVID-19. While physician practice guidance is available from many sources, the AesCert Guidance has been developed specifically for aesthetic medicine settings. In a number of areas, this Guidance exceeds traditional aesthetic office safety precautions, recognizing reduced tolerance in an elective treatment environment for any risk associated with COVID-19's highly variable presentation and unpredictable course. The disease has placed a disturbing number of young, otherwise healthy patients in extremis with severe respiratory and renal failure, stroke, pericarditis, neurologic deficits and other suddenly life-threatening complications, in addition to its pernicious effects on those with pre-existing morbidities and advanced age. Accordingly, the Guidance seeks to establish an elevated safety profile for providing patient care while reducing, to the greatest extent reasonably possible, the risk of infectious processes to both patients and providers.

While the Guidance categorically cannot foreclose the risk of infection, nor serve to establish or modify any standards of care, it does offer actionable risk-mitigation considerations for general office comportment and for certain non-surgical procedures typically performed in aesthetic medical settings. This Guidance is purely advisory in nature and should be regarded as a set of baseline precautions that should be considered; however, it is not an exhaustive list of everything required to operate safely. It is axiomatic that all such considerations are necessarily subject to the ultimate judgment of each individual healthcare professional based on patient situation, procedure details, office environment, staffing constraints, equipment and testing availability, and local legal status and public health conditions.

Importantly, this Guidance is also subject to present limitations on medical and scientific understanding of COVID-19, and any future changes in such understanding will need to be evaluated by providers in determining its continuing utility. Additionally, this Guidance has been prepared in a nationwide environment marked by limited diagnostic resources for both active disease and possible immune response, and an absence of validated pharmaceutical treatments or vaccines. As point of care testing becomes more widely available, affordable and reliable, and once therapeutic or preventive protocols are in place, such developments may permit certain modulation of the Guidance.

In the interim, federal, state and local government legal pronouncements and public health conditions will inform the gating decisions of when it is permissible and prudent to reopen practices and re-engage with patients, and whether to limit certain procedures which may present greater contagion risk. Given the multiplicity of such circumstances across the country, these are necessarily highly localized and indeed individualized assessments. While such gating decisions are not the focus of this Guidance, it seems clear that practices should consider, at a minimum, whether in their local communities: (1) new COVID-19 cases are declining sequentially to eliminate or at least substantially control community spread, (2) testing is available at meaningful scale to validate perceived prevalence reductions, and (3) adequate protocols and resources are in place in conjunction with local health departments to conduct effective contact tracing where necessary in response to COVID-19 incidents. Without robust testing, the ability to effectively identify individuals with COVID-19, do appropriate tracing, and isolate and treat the infected is substantially reduced. Therefore, in the absence of these enumerated local conditions, practices must cautiously factor the attendant increased risk of transmission into their reopening calculus.

Further, subsequent to the threshold decision to reopen, it is possible that future COVID-19 prevalence in a particular community, along with limits on testing and treatment availability, could periodically require limitations in scope of practice or even temporary office closure to reduce risk of harm. Again, this Guidance takes no position on these contingencies, and seeks only to provide information and best practices for operational implementation where it is otherwise legally permissible and medically responsible to interact with patients in the office setting for delivery of medical aesthetics care. More broadly, in this highly dynamic pandemic environment, this Guidance is necessarily based on, and its applicability confined to, the public health environment and related government pronouncements in effect as of the date of publication. Subsequent evolution in transmission prevalence, testing and tracing capacity, and treatment as well as vaccine availability could warrant either further restriction or expansion of aesthetic practice from this Guidance, depending on the direction of such evolution.

In the meantime, based on the current public health landscape and the medical and scientific information now available, the Guidance next proceeds to outline a series of practical considerations associated with practice reopening, ranging from preparing the medical office environment, staff training, and patient and staff health screening, to treatment room set-up, selection of Personal Protective Equipment (PPE), and precautions for common office aesthetic procedures, such as neurotoxin and dermal filler injections, energy-emitting devices, body contouring and medical skin care treatments.

### III. Medical Office Preparedness

#### Patient communication and transparency

While effective patient communication and transparency are always a hallmark of any well-functioning medical practice, they are particularly critical during the return-to-work phase of this COVID-19 outbreak. Accordingly, it is important for practices not only to implement and follow high safety standards as a substantive matter of public health, but also to clearly convey these steps to their patients to foster a sense of awareness and confidence. Therefore, as an overarching theme to this entire Guidance, measures and changes undertaken by practice in response to the COVID-19 outbreak should be proactively signaled to patients to heighten confidence. Utilizing established means to communicate to patients, such as the practice's website and notifying patients via digital and direct personal communication, is an important first step in conveying the practice's commitment to the health and safety of patients and the public, while maintaining high-quality patient care.

Communicating new policies and protocols throughout the clinic with visible reminders such as display posters and other signage will assist staff in remaining vigilant, in addition to conveying a practice's emphasis on safety for patients and others. It is advisable to display such materials throughout the clinic, including common areas, reception areas, waiting room, treatment rooms and bathrooms, reminding patients and staff of symptoms related to COVID-19, healthy hygiene and prevention etiquette. Examples of display posters are provided in the following links, although it should be noted with respect to the symptoms poster that this is not an exhaustive list, and additional symptoms are increasingly recognized, such as severe fatigue, nausea and diarrhea, chills, repeated shaking with chills, myalgia, headache, sore throat, new loss of taste or smell, and unexplained anorexia: symptoms of COVID-19 – sample poster/flyer, preventing the spread of COVID-19 – sample poster/flyer, and handwashing and respiratory hygiene – sample poster/flyer.

Additionally, in the same spirit of patient transparency and disclosure, given that there remains inherent, ineliminable risk of an infectious process or other complication arising from any sort of medical procedure during an ongoing global pandemic, even after respecting the considerations set forth in this Guidance and elsewhere, practices may wish to append a COVID-19 disclosure to their standard patient consent form. An example consent form developed by the American Society of Plastic Surgeons (ASPS) may be found using the provided link.^16^ Further, in the case of patients at higher risk of COVID-19 complications, such as those who are of advanced age, immunocompromised, or otherwise afflicted with cardiac or respiratory conditions or other comorbidities such as diabetes, hypertension or obesity, consideration should be given to possibly delaying aesthetics intervention, if patient risk factors are deemed too high.^19^

#### Managing the office environment – general guidance

It is clear that during this pandemic, social distancing (hereinafter referred to as “physical distancing” in order to emphasize the intended minimum physical separation of six-feet between individuals, and limits on congregating in large groups) is as important to the safe operation of a medical aesthetics practice as to any other business, household or community. And it will remain so for the foreseeable future. Accordingly, as a second overarching theme running through this Guidance, physical distancing principles should be incorporated throughout the practice, from the moment of initial patient scheduling through post-procedure check-out, and all office workflows from staff's arrival in the morning until the doors are locked at night. Put simply, limiting the number of individuals in a particular setting and space at a given time is fundamental to minimizing transmission.

Utilizing telemedicine and leveraging remote videoconferencing technology for patient consultations and non-procedure visits will aid in minimizing office traffic while allowing for the development of a treatment plan (both short- and long-term), building rapport with patients and reducing in-office contact time.^2,16,17,20^ The efficiency and value of this approach can be enhanced by sending patients a pre-consultation form in advance of a scheduled telehealth interaction to learn more about patient's primary concerns and the type of information or treatment they are seeking. Capturing this information in a more formal manner will help facilitate and guide discussion between the patient and clinician in a manner that timely surfaces various opportunities to divert in-person visits to safer, more efficient interactions. Various enabling developments have occurred in this regard, ranging from the proliferation of telehealth and other video technology platforms, to certain applicable standards surrounding the privacy and reimbursement of distance versus in-person provider interactions (N.B. - legal requirements vary by jurisdiction).^20^

With respect to treatment-related visits or other necessary in-person office visits, consider spacing or staggering appointments to reduce the number of patients in the office at one time and to allow for proper disinfection between patients. Mindful of office size and staffing constraints, consider limiting overall patient volume per day, or extending office hours to spread patients out over a longer time horizon throughout the day.

Remind patients of the need to arrive to their appointment promptly and alone, and that individuals accompanying patients will be required to wait in their vehicle or outside of the office for the duration of the appointment.^2,16^ Special arrangements may be made in advance for minors, elderly patients or persons with disabilities. Visitors spending any time in the office should be screened in the same manner as patients. And remember for any staff, patients or other visitors in the office, it is important to observe and model safe physical distancing by limiting greetings to a smile, wave and other non-contact gestures.

For treatment interactions, endeavor to limit the number of staff members in the treatment room during procedures. For example, where possible, consider whether a sole provider is able to appropriately perform a particular procedure without the need for other staff in the treatment room. If not, consider allowing the provider to be accompanied in the treatment room by no more than one medical assistant, with such staff person observing safe physical distancing across the room as circumstances permit. Develop and adopt policies regarding fellows, residents, medical students, and visitors to reduce to an absolute minimum the number of individuals in any given treatment room and the office as a whole. Training opportunities for staff and residents should be subordinated to the requirements of physical distancing, and this safety measure may be worth expressly messaging to patients, who may appreciate understanding that the practice has taken measures to prioritize their health.

Keep treatment room doors closed and utilize place card signage on treatment room beds or chairs to inform patients that rooms, beds, chairs, surfaces and instruments have been disinfected. Remove unnecessary blankets, pillows, robes or headbands from treatment rooms, and limit items on countertops.

Reorganize waiting rooms by either removing chairs and spacing the remaining chairs at least six feet apart, or by designating certain chairs to be used and others not to be occupied. Consider limiting the size of the waiting room or common areas to create “natural” barriers (e.g. potted plants, tables) to prevent individuals from congregating in one area. Remove magazines, promotional or other collateral reading materials from the waiting room, treatment room and reception areas. Patient reception coffee, beverage and snack bar service should be discontinued. For the occasional patient who might require food or drink for a medical condition, such as a diabetic who develops hypoglycemia, especially when instructed to be NPO pre-procedure, items can be provided as needed from storage. And importantly, place alcohol-based hand sanitizer, hand wipes and tissues, with no-touch trash cans, liberally throughout the clinic, accessible to both patients and staff.^2,16^

In managing the movement of patients and others through the office, consider limiting points of entry and exit, strive for one-way traffic in hallways where possible, and try to designate separate areas for patient screening and check-in, as well as check-out. Care should be taken to ensure any such special ingress/egress restrictions do not violate applicable building codes and can be overridden to permit safe evacuation in case of emergency.

If possible, take patients upon arrival directly to treatment rooms for screening and check-in, in order to limit or entirely eliminate people congregating in the waiting room. Ideally, check-out could be handled the same way, and for all patient administrative paperwork at check-in and check-out, clean and disinfect clipboards between each use, and consider providing single-use disposable pens to avoid multiple individuals handling the same writing instrument. For the same reason, if possible, encourage the use of remote payment systems instead of credit cards and cash, in order to minimize touching of credit card machines and office tablets.

Staff seating and work-stations should be reconfigured to respect physical distancing. For internal collaboration, staff should employ one-on-one or small meetings (depending on space availability) to allow for appropriate safe interpersonal distancing or arrange for virtual meetings.

Alert all vendors and contractors regarding new office policies limiting the number of visitors to those that are integral to either clinical practice or the business functions of the office. Additionally, insist that vendors and contractors be aware of and follow the clinic's “stay home if sick” policy. Direct all delivery personnel to a designated area for drop-off of packages and proper package disinfecting.

#### Cleaning and Disinfecting Practices

In additional to proper physical distancing, the cleaning and disinfecting practices that are part of any medical aesthetics practice in ordinary times should be elevated and sustained during this period. First, prior to reopening a practice to patients, a qualified professional cleaning service should conduct an initial, comprehensive deep cleaning and disinfecting of the entire facility. A professional service should similarly perform a thorough cleaning and disinfecting process following the close of business each workday.

On a regular basis throughout each workday, the entire staff should be trained on and committed to ongoing cleaning and disinfecting roles. Based on the transferrable nature of COVID-19, enhanced frequency of disinfecting surfaces throughout the day and between patients is critical in protecting the health of patients and staff members.^**5**^ Developing a protocol and cross-training individuals responsible for managing and monitoring cleaning may be helpful in the adoption and consistent execution of these new processes. Creating a checklist and schedule and displaying it on treatment room doors can serve as a reminder for staff and demonstrates to patients that treatment rooms are being consistently supervised and disinfected before their particular treatment (Appendix Figures A1 and A2).

With the aforementioned frequency, cleaning should also include disinfecting all common, high-touch areas such as the waiting room, reception areas, check-in and check-out areas, kitchen and break rooms, labs, offices and workstations, computer keyboards, tablets, credit card machines, pens and bathrooms (Table 1). And again, at the end of each workday, a thorough, supervised professional cleaning service is an essential daily practice.

**Table 1.** **Minimum Necessary Areas for Cleaning and Disinfecting**

When disinfecting surfaces, staff should wear disposable gloves and any additional protection based on the cleaning products being used and the potential risk of exposure. Use of 70% ethyl alcohol is recommended in disinfecting small areas, or discrete items between repeated use such as reusable dedicated equipment (e.g. thermometers). Environmental Protection Agency (EPA) registered disinfectants include the use of sodium hypochlorite at 0.5% (equivalent to 5000 ppm) for disinfecting surfaces, as well as a range of other common cleaners such as Clorox disinfectants containing either sodium hypochlorite or quaternary ammonium; Lysol products containing sodium hypochlorite, quaternary ammonium, hydrochloric acid, or citric acid; and Purell ethanol-based products.^2,13,16,21^ The list of disinfectants that meet EPA criteria for use against SARS-CoV-2 may be found in the link provided here.^21^

Steps to properly disinfect surfaces include first cleaning an area or item with soap and water or another detergent prior to using a proper disinfectant. In addition to recommended use of EPA-registered disinfectants, make sure rooms are adequately ventilated and follow label instructions, as some products recommend keeping surfaces and items wet for a period of time to enhance antimicrobial activity. It is recommended before donning and immediately after doffing gloves to wash hands thoroughly or use an alcohol degerming solution (hand hygiene solution). CDC steps for cleaning and disinfection may be found here.^13^

#### Personal protective equipment and medical supplies

Adequate PPE (principally, face masks, gloves, gowns, and goggles, shields or other eye protection) are necessary to protect providers and serve to protect patients alike, and therefore should be viewed as indispensable for reopening, and continuing to operate, any medical aesthetic office. This Guidance recognizes the lamentable reality that severe PPE shortages on a global scale continue to pose significant challenges to the entire U.S. healthcare system at the time of Guidance publication, and in some cases the impact of this shortage on particular practices has been exacerbated by those practices' decisions during the last several months to contribute PPE inventory to hospitals, emergency rooms and first responders in their respective communities. Notwithstanding such supply chain challenges, however, adequate access to and deployment of PPE within a practice, both initially and on an ongoing basis, should be viewed as a precondition to medical office and patient care activity. It is imperative that practices proactively develop a plan to optimize their supply of PPE, both for current needs and in the event of future shortages, and to identify mechanisms to procure additional supplies when needed. Continually assessing quantities and replenishing supplies throughout the day, as well as monitoring public health agency recommendations regarding the use of PPE for healthcare professionals, are critical as the COVID-19 outbreak evolves and public health guidance shifts.

The type of face mask and other PPE recommended for use by healthcare professionals is based upon anticipated risk of exposure to COVID-19 while performing specific procedures.^4^ More broadly, Occupational Safety and Health Administration (OSHA) recommendations regarding the type of PPE used by a healthcare professional or individuals working in a healthcare setting are based upon anticipated risk of exposure while performing specific tasks or procedures (Table 2).^2,3,4^

**Table 2.** **OSHA Occupational Risk Exposure Levels**

Starting from this framework and given the current state of the COVID-19 crisis, this Guidance recommends that practices should consider a strategy of having every employee in the office, irrespective of function, wear a three-ply surgical mask. Given existing supply chain limitations, if it is impossible to source such three-ply surgical masks for an office's clerical or administrative employees who have no or minimal patient interaction, a professionally-manufactured cloth mask with full mouth and nose coverage is preferable to no protection; however, this Guidance deems a three-ply surgical mask to be the recommended practice for all staff in the current environment.^16^ For reasons of both substantive protection and patient confidence, handkerchiefs, scarves and other homemade masks should not be utilized by any staff, and if inventory constraints require that these are the only option, practices should seriously consider the wisdom of having such employees on-site at all. It is recommended that surgical masks used by clerical or administrative staff be discarded and replaced on at least a daily basis. To the extent such employees are permitted by an office to utilize cloth masks, they should generally be deployed for no longer than one day without being professionally laundered prior to next use.

Providers or staff who are involved in administering treatment or are otherwise in the treatment room for any patient assessment or general treatment or care should wear, at a minimum, a three-ply surgical mask, eye protection in the minimum form of safety glasses, and gloves. Inventory permitting, use of a gown is also recommended for all such individuals associated with the treatment room. Masks, gloves and gowns involved in treatment should generally be considered single-use and safely disposed after each patient procedure, and eye protection should be cleaned and disinfected with the same regularity.

For those providers or staff performing, assisting with, or otherwise in the treatment room for any Aerosol-Generating Procedures (AGPs) as described more fully later in the Clinical and Non-Surgical Treatment section of this Guidance, the use of an N95 filtering face piece respirator (N95 mask), or its equivalent, is the minimum nose and mouth protection required, providing respiratory protection and protection from blood and body fluids.^17^ For such AGPs, in the absence of N95 availability, OSHA indicates other types of acceptable respirators with similar or greater protection may be used, such as R/P95, KN95, N/R/P99, and N/R/P100. These respirators are often more comfortable for the wearer when fitted with a valve exhalation feature, but this feature has the effect of elevating wearer safety over that of patients and others in the vicinity, and therefore is generally discouraged. Single-use gloves and gowns should also be used for all providers and staff in the treatment room for AGPs, and providers administering treatment should consider use of a single-use surgeon's cap. Further, providers administering AGPs should consider using heightened eye protection beyond standard safety glasses, such as full goggles or face shields, which should be thoroughly cleaned and disinfected after each use. Notwithstanding any inventory challenges, N95 masks used in AGPs should generally be considered single-use only, unless used in conjunction with a face shield and proper disinfection procedures are utilized (Table 3). The CDC generally recommends use of a cleanable face shield over an N95 when feasible.

**Table 3.** **Aesthetic Procedures and Treatments-Protection and Disinfection**

Non-AGP procedures performed above the clavicle generally pose greater risk than lower body procedures, and therefore practices should consider whether higher level PPE items should be utilized.^16^ For non-AGP procedures involving the head and neck region, particularly where detailed work requires the provider to remain in close face-to-face proximity with a patient's airways, use of an N95 mask and more substantial eye protection is preferable.

Irrespective of job function or procedure type, PPE training should be provided to all providers and staff throughout the office and across job function, including proper hand hygiene practices, correct fit, donning and doffing to avoid cross-contamination, and considerations for contemplated extended use or reuse of PPE.^4,22^ In particular, users of N95s or other respirators should be fit-tested prior to first use, thereafter on an annual basis, or more frequently in the event of significant weight loss or change in facial hair. The CDC fact sheet on use of PPE and proper donning and doffing may be found here.^22^

Due to PPE shortages, certain medical societies and other public health authorities have advised that healthcare professionals who typically wear a mask for procedures may consider wearing the same mask throughout the day in an effort to conserve PPE.^2^ For example, the CDC has provided guidance on practices allowing the extended use and limited reuse of N95 masks when supplies are depleted. Extended use (leaving the mask on for multiple patient encounters, without removal) is generally favored over reuse (using the same mask for multiple encounters and removing it between encounters), as there is less risk of repeat handling-related contact transmission. In addition to strict adherence to proper hand hygiene practices before and after touching or adjusting the mask, proper fit and function are paramount to safe extended use or reuse.^15^ Manufacturers may have specific guidance regarding reuse. However, variables such as contamination over time make it difficult determine the maximum number of reuses. Ultimately, single use of a mask is lower risk than extended use or reuse, and therefore reflects the general consensus of this Guidance, particularly for AGPs, unless, as stated above, a mask is used underneath a face shield and proper disinfection of the face shield occurs after each procedure.

The other variable here involves the highly dynamic state of PPE decontamination technology. For example, certain hospitals have been developing vaporized hydrogen peroxide systems for decontaminating N95 masks, and studies are beginning to validate various other modalities and protocols as well.^23^ Accordingly, it is impossible for the Guidance to anticipate and adjudicate every permutation of PPE deployment duration and decontamination, for example, the possible single use of a surgical mask over an extended use N95 for a non-AGP procedure. Such decisions are necessarily subject to the best professional judgment of the provider on a case-by-case basis, based on the specific combination of patient and procedure risk factors and the overall PPE availability and decontamination landscape.

If extended use or reuse of face protection is necessary, take care to avoid touching the mask or respirator itself, touching only the fasteners when donning and doffing. If reuse across multiple days is unavoidable, it is incumbent upon providers to ensure thorough decontamination and safe storage. Such contingencies are beyond the scope of the position taken by this Guidance, but it is observed that thoughtful procedures have been articulated elsewhere, ranging from a study on a proposed N95 decontamination protocol,^23^ to a proposal for cycling five masks over a five-day period, using one mask per day and then storing each individually until the same day the following week to allow for a seven-day period of non-use of each mask. If attempted, any such storage should be in a sealed, breathable container between uses to reduce damage, labeled to identify user, date and duration of prior use, and with thorough disinfecting or disposal of containers on a regular basis.^15^

Beyond mouth and nose protection, wearing protective eyewear in conjunction with a mask when treating patients reduces exposure and inadvertent touching of facial mucous membranes. With respect to eye protection, prescription eyeglasses do not afford adequate protection in the COVID-19 treatment environment, without wearing a wraparound style of secondary eyewear. For AGPs in particular, full wraparound goggles are recommended, again with a face shield advisable as well. Special attention should be paid throughout the office, and not just the treatment room, to those wearing prescription eyeglasses or non-prescription readers, as plastic or metal surfaces have the potential to become a fomite for COVID-19 given the propensity of frequent touching of one's eyeglasses throughout the day. As with staff cell phones, eyeglasses should be cleaned and disinfected throughout the day, and staff should avoid touching or handling such objects between hand washings.

As a corollary to the need for consistent use of PPE, there are emerging reports of skin complications resulting from repeated PPE exposure and excessive hand hygiene, especially among healthcare workers. These complications variously include skin breakdown, erythema, papules, scaling, burning, itching and stinging. Providers may wish to consider proactive and therapeutic use of emollients, barrier repair creams and other skin calming and hydration preparations to mitigate such conditions.^24^

In addition to PPE, other medical supplies of particular import to managing the COVID-19 environment include alcohol-based hand sanitizer and hand wipes, which should be placed at entry and exit points and throughout the office including in the waiting area, reception area(s), treatment rooms, and bathrooms. Ensure the availability of liquid soap at sink areas, and facial tissues and no-touch wastebaskets with disposable liners and lids throughout the office. Non-contact thermometers (infrared or thermal scanner models) are recommended in lieu of forehead, oral or tympanic (auditory canal) thermometers.^10,11^

### IV. Employee and Patient Health

#### Employee health and training

In order to offer safe care in a safe environment to their patients, practices must first ensure that providers and staff are healthy and do not constitute a transmission vector. This starts with clearly and proactively communicating to all employees the clear mandate to stay home if sick or experiencing any early suggestion of symptoms, and reviewing applicable benefits and provisions related to sick leave, caring for sick family members and children, and flexible scheduling. Questions and concerns regarding employee health, safety, compensation and benefits may arise, and are heightened during these uncertain times. Information should be provided about available employee assistance services and steps employees can take to protect themselves at home.

With respect to COVID-19 in particular, practices should develop an infectious disease preparedness plan that addresses the level of risk associated with various jobs and tasks to help guide actions and reduce the risk of employee exposure to COVID-19. Assessments should be undertaken of potential sources of employee exposure to COVID-19, including coworkers, patients, the general public, individuals that are symptomatic or who have recently been symptomatic for COVID-19 or a febrile respiratory tract infection, and those at high risk (e.g., other healthcare workers, travelers who have visited locations with widespread COVID-19 transmission, including domestic locations with significant community spread, etc.). Attention should be given to non-occupational risk factors at home, including family and immediate or close contacts, and community settings (e.g., attendance at recent large gatherings or events) and individual risk factors (e.g., immunocompromised status and various chronic conditions).^4^

As part of this larger process, an employee health screening should be completed every day before staff enters the office or beyond a designated assessment area. Models vary, but at a minimum this could be accomplished with a short form, or even an email to a designated responsible person in the office, constituting a quick self-attestation that an employee is asymptomatic and otherwise unaware of any exposure to a confirmed or suspected COVID-19-positive individual, with such records being maintained by the practice in either paper or preferably digital form. Even better, if feasible, a daily employee wellness check should be performed in addition to the symptoms self-report, comprised of a temperature check using a non-contact thermometer, and any necessary follow-up.

Any employee who reports feeling sick, senses any early hint of symptoms, or exhibits elevated temperature or other symptoms is required to refrain from entering, or immediately leave the office and follow up with their primary care physician or other appropriate offsite care facility for evaluation and, as indicated, viral testing. Depending on the nature and extent of any positive findings and follow-up testing, office policy should dictate a protocol for minimum time off work and appropriate timing of return based on symptom progression, cessation, and all test results. It is advisable to select one or two individuals (“Workplace Coordinators”) in the practice to serve as point persons in this regard, and more generally for all COVID-19-related issues in the practice, including oversight of clinic infection prevention measures. Establish this communication plan early, clearly communicate and share it with all employees.

Beyond this initial screening and preparedness plan, as a general matter, all employees should model physical distancing and good hygiene practices in all office activities, whether related to patient interactions or otherwise. This includes minimizing use of shared workspaces, office supplies and medical instruments, such as sharing other employees' phones, desks, offices, computers and other equipment.^5^ Staff should minimize handling of personal cell phones throughout the workday, and refrain from any cell phone handling between their last hand washing and any patient contact. Lunch rooms and staff lounges should be closed or restricted to limited size and spaced groups and alternating schedules. With respect to hygiene, all employees should engage in frequent, thorough handwashing (for at least 20 seconds) and cough and sneeze etiquette.^18^ The World Health Organization (WHO) counsels healthcare professionals to follow “My Five Moments for Hand Washing,” using alcohol-based hand sanitizer or soap and water: (1) before touching a patient, (2) before engaging in clean or aseptic procedures, (3) after potential exposure to body fluids, (4) after touching a patient, and (5) after touching patient surroundings.^7^

To keep employees safe, as previously discussed, it is advisable to consider use of surgical masks by all staff regardless of job function; a minimum of surgical masks, protective eyewear, gowns and gloves for all staff involved in any procedures; and the addition of N95 or equivalent masks, more fulsome eye protection and/or a face shield, and possibly a surgical cap for all staff involved in AGPs. Again, effectiveness of PPE is highly dependent on proper handling, fit, and correct and consistent use; therefore, employee training on these topics is critical. And in addition to the aforementioned WHO handwashing moments, handwashing is also required before putting on, after taking off, and whenever touching or adjusting PPE, always careful to handle face protection only by the fasteners without touching the mask itself.^18^ More generally, employees should avoid touching their eyes, nose or mouth with gloves or bare hands, both in connection with PPE use and otherwise around the office and throughout the workday.

In addition to PPE, staff clothing decisions bear on office safety and patient confidence. To avoid the risk of clothing as a transmission vector into or out of the office, it is recommended that surgical scrubs or other dedicated office uniforms be worn by all providers and staff, even those who are not in immediate proximity to patients. When practicable, these should be worn only in the office, not commuting to and from work, changed daily, and thoroughly laundered by a professional service that collects soiled garments from the office to avoid employees bringing dirty laundry home and risking cross-contamination.

#### Recognition of symptoms

It is vital that all providers and staff, including those who serve in non-clinical patient contact roles such as receptionists and other administrative personnel involved in patient scheduling, check-in or check-out procedures, be able to identify and report the symptoms associated with COVID-19. Symptoms may range from mild to severe and appear anywhere from approximately 2–14 days following exposure. Symptoms of COVID-19 to be on alert for include flu-like symptoms, fever (≥100.4°F or 38°C), cough or shortness of breath, new nasal congestion or runny nose, loss of taste or smell, as well as non-specific symptoms such as sore throat, myalgia, fatigue, nausea and diarrhea. Additional symptoms reported include chills, repeated shaking with chills, muscle pain, and headache.^9^ All employees should also be trained to identify emergency warning signs that require immediate medical attention, such as trouble breathing, persistent pain or pressure in the chest, new confusion, inability to arouse a patient, and bluish lips or face.^6^

Employees should be vigilant for all these symptoms, not only in themselves and coworkers in the office, but also patients, vendors, contractors and other visitors to the office. At any sign or suspicion of COVID-19 symptoms, the affected individual should be required to refrain from entering, or immediately leave, the office, and a Workplace Coordinator should be promptly notified, in a HIPAA-compliant manner in the case of patients.

#### Symptomatic patients and employees

With respect to prospective patient visits, appointment scheduling processes should be modified to include pre-screening patients before their office visit in conjunction with appointment reminder calls to help identify potential infection and recent risk of exposure.^3^ A phone screening tool may be developed for use as a wellness checklist in this regard to aid in surfacing any areas of concern (Appendix Figure A3).

If through such telephonic pre-screening efforts patients report symptoms potentially associated with COVID-19 or have indicated potential sources of exposure by other means based on recent contacts or travel, explain to patients that out of an abundance of caution, they will need to reschedule their appointment for a later time. In the event of symptoms, also recommend they promptly follow up with their primary care physician or other appropriate offsite care facility for evaluation and, as indicated, viral testing. The minimum timeframe for rescheduling any such patients is a risk-based assessment depending on symptom presentation and testing results and should be governed by applicable CDC guidelines.

In addition to patient pre-screening, offices must implement a protocol for health screening patients immediately upon arrival on the date of appointment, ideally in a contained area designated for this purpose to minimize other interactions prior to clearance (Appendix Figure A3). As with employee health screening, this process should include both a form of questionnaire for eliciting disclosure of symptoms or other exposures, and a staff-administered temperature check using a no-touch thermometer. If on the appointment date a patient presents and reports symptoms or exposure to known or suspected COVID-19-positive individuals, or if temperature check or other assessment by staff reveals that a patient is symptomatic or at high risk upon arrival, immediately isolate the patient in an unoccupied room, provide a surgical mask for the patient to apply, irrespective of whether the patient arrived uncovered or over whatever mask with which the patient arrived, minimize contact with others in the clinic, and quickly and discretely remove the patient from the office. If the patient is well enough to drive home, send the patient home immediately. Recommend that patients isolate themselves at home, practice careful infection prevention measures, and follow up immediately upon returning home with their primary care physician or other appropriate offsite care facility for evaluation and, as indicated, viral testing. Patients may be alarmed and anxious to discover that they may have symptoms or are otherwise at risk of prior exposure related to COVID-19. Remain calm and supportive of the patient and continue to adhere to predefined infection prevention protocols. Depending on the nature and extent of the patient's positive follow-up findings and testing, office policy should dictate a protocol for minimum time away from the office before a rescheduled office visit may be permissible, based upon the patient's symptom progression, cessation, and all test results.

This same isolation-and-exit protocol applies equally to employees, who despite having presumably observed the practice's stay-home-if-sick policy, may first become symptomatic at work, or otherwise stimulate a positive finding during the previously described employee arrival screening process. The procedure would similarly apply to any vendors, contractors or other visitors to the office, all of whom should be notified to stay away if sick or at risk, and then subjected to a similar screen-on-arrival protocol. In the event of a positive visitor screen, the same isolation-and-exit protocol obtains.

Following execution of the isolation-and-exit protocol, in the event of any on-site presence, however brief, by any patient, employee, vendor, contractor or other visitor who is either suspected or confirmed to have COVID-19, it is imperative to immediately switch focus to minimizing risk to the office premises. In this regard, care should be taken to follow applicable infection control guidelines, and thoroughly clean and disinfect all area(s) the individual had accessed or moved through.^12^ The CDC recommends closing off all areas accessed by that individual to reduce intra-office contamination, opening external doors and windows to that contained area to increase air circulation from the outside, and (if feasible) waiting 24 hours before cleaning and disinfecting to minimize potential exposure of others to respiratory droplets.^12,14^ Ideally a professional service should be utilized for this reactive cleaning, and any staff involvement should require use of adequate PPE.

Depending on the nature, duration and overall extent of the individual's activity in the office, including interactions with other patients, employees and others, the practice may need to consider further prophylactic measures up to and including temporary office closure, in order to minimize further exposure, ensure adequate site remediation, and assess the risk of further transmission. Finally, it is advisable to notify all patients, employees and others who may have been exposed to any such known or suspected COVID-19-positive individual in the office.^17^ State and local laws and public health regulations, as well as other canons of professional responsibility, are likely to govern or otherwise inform these disclosure obligations, and accordingly such contact tracing ought to be undertaken in coordination with local health departments and other authorities.

### V. Patient Management Procedures

#### Pre-screening patients

Much of the physical distancing-related protections that a medical aesthetics practice can leverage to enhance COVID-19 safety are a function of decisions made before a patient ever arrives at the office. Thoughtful pre-screening procedures and advance communications serve to limit the need for office visits and minimize the duration of and unnecessary contacts during those that do occur. Patients should be educated on how these new measures have been implemented to enhance their safety, and what they should expect when they arrive.

As discussed previously, consultations and other non-treatment appointments may be arranged through patient portals, telemedicine or other technology-enabled communications.^2,16^ Billing and other administrative matters, treatment plans and other preparatory items can be addressed over the phone or by video-conference, thereby shortening office stays.

#### Office arrival, check-in and check-out

Patients should be encouraged to arrive to their appointments alone, and notified that individuals accompanying them will be required to wait in their vehicle or outside the office for the duration of the appointment.^2,16^ Special arrangements can be made for the elderly, minors and persons with disabilities.

From their vehicle, an arriving patient may call or text the contact number for a designated HIPPA-trained staff member and wait until the staff member indicates the patient may enter the office. Staff should greet each patient at the entrance to guide them through the intake process and to an appropriate location. A patient screening flow chart may assist staff in mapping this and subsequent steps in a patient arrival process designed to combine heightened safety protocols with efficient and responsive customer service (Appendix Figure A4).

Patients should be advised to bring a face mask or similar covering with them to their appointment, and informed that they will be required to wear it for the duration of their appointment, to be removed only if and to the extent they are undergoing facial procedures.^16^ In the event patients forget or are unable to bring a face mask, they should be provided one for use throughout their appointment. While a three-ply surgical mask is ideal and will inspire elevated patient confidence, limitations on PPE availability would alternatively justify providing another form of commercially-manufactured cloth mask instead. Staff should remind patients not to adjust their face mask or touch their eyes, nose or mouth, and that if they must do so they will need to wash or sanitize their hands before and after such contact.^8^ Similarly, patients should be counseled to minimize handling their cell phones during the appointment, and to rewash or re-sanitize their hands following any such use.

For those practices located in multi-story buildings serviced by elevator access, patients should be counseled on best practices for elevator use in this environment, beginning with the need to arrive early to allow extra time to wait for a less crowded elevator that permits physical distancing. Once inside, maximum spacing from other riders should be sought, facing forward, and ideally as close to the front of the elevator, and hence the doors, as possible for access to outside air during any intervening stops. Patients should wear their mask at all times, and avoid touching elevator buttons with bare hands, instead using a clean tissue, elbow or other similar approach. Alternatively, if elevator circumstances appear to defy safe usage and stairs are a viable option given the office's floor location and the patient's physical capacity, it may be useful to provide the location of applicable stairwells.

Upon arrival in the office suite, consider having designated staff take the patient directly to an exam room for check-in, in order to avoid congregating in the waiting room or the common area around the reception desk. Confirm the patient has already donned a mask, request the patient wash or sanitize their hands, and then proceed to conduct a wellness assessment to confirm the absence of a fever, other COVID-19 symptoms or related high-risk exposures. A similar form of the wellness screening checklist used at the prior time of telephonic appointment confirmation may be repurposed at the time of office arrival (Appendix Figure A3).

Following the wellness screening, it is advisable to endeavor to complete the patient's ensuing aesthetic services with minimal relocation throughout the office, preferably in the same treatment room in which check-in occurred if possible, or alternatively in such other manner as reduces the patient's geographic footprint and multiplicity of interactions within the office.

Further, it is recommended that, if possible, patient check-outs be conducted within the treatment room, or an otherwise designated, separate check-out area to avoid re-exposure to reception or other common areas.

Finally, the office should conduct a post-visit follow-up video-conference or telephone call to the patient several days after the appointment, both to monitor progress post-procedure and also to ascertain whether any COVID-19 symptoms have recently developed despite the patient having been asymptomatic at the time of the appointment. Here again, a wellness screening checklist may be a useful tool for staff (Appendix Figure A5). Any positive report during this follow-up may trigger contact tracing considerations and other remedial measures by the practice. Further, even if patients report being asymptomatic at this follow-up, they should be asked to notify the office in the event they subsequently develop any COVID-19 symptoms within the balance of the remaining 14-day period of their recent appointment, again in order to permit appropriate contact tracing.

### VI. Clinical and Non-Surgical Treatment-Related Guidance

#### Treatment room set-up

Due to the duration and proximity of patient and other interpersonal contact, as well as the possibility for various procedure-specific activities to elevate the risk of viral shedding, the treatment room requires particularized attention to safety concerns and practices. For containment purposes, doors to treatment rooms should remain closed during and in-between use. Office-wide air handling systems should be evaluated to understand the path and extent of circulation of air from the treatment room vents into other rooms and common areas throughout the office, and where possible, to minimize such flow. Where available, external windows may be opened during inter-procedure treatment room cleaning to provide maximum ventilation. Thorough cleaning and disinfecting of treatment rooms and all exposed surfaces and equipment, whether or not utilized in the prior procedure, must be performed after each patient.

Patient visits often involve more than one type of procedure during a scheduled appointment (e.g., neuromodulator injections and dermal fillers). Where possible in view of device and other equipment (including digital photography or camera systems) deployment throughout the office, consider consolidating multiple patient treatments into a single treatment room to minimize multiple points of exposure. Further, it may be advisable to consider limiting the number of procedures or grouping the type of procedures per patient visit in order to reduce multiple patient exposures, contact time and overall appointment duration.

In advance of a patient procedure, it is advisable to take all steps necessary to prepare equipment, supplies and other positioning of assets prior to bringing the patient into the treatment room, in order to minimize exposure time between the staff and patient. Examples of advance planning in this regard includes preparing all trays, instruments, supplies, drugs, and injectables. In the case of energy-based devices, this might include turning the equipment on and pre-performing setup tasks, including calibration to the treatment parameters if known for the specific upcoming case. Sterile items should be left in packaging to be opened in the patient's presence, both for safety reasons and to instill patient confidence. It is important to train staff on, consistently follow, and consider visibly displaying confirmation of, a treatment room cleaning and disinfecting protocol and schedule in each room, again both to ensure substantive office compliance and to promote patient confidence (Appendix Figure A1).

Additionally, as previously reported elsewhere in Sections III and IV of this Guidance, it bears reemphasis that PPE is of particularly critical import within the treatment room during this time, for reasons of safety, patient perception and the overall risk minimization required to justify elective aesthetic procedures during the current phase of the COVID-19 outbreak. Accordingly, this will result in recommended use of masks, gowns and protective eyewear in certain procedures where many healthcare professionals previously may have justifiably used none, and heightened PPE protocols across a number of other procedures beyond what was previously the norm.

#### Anesthesia and analgesia

When topical anesthetic agents are used for office-based aesthetic procedures, it is common for application time to range from approximately 30–60 minutes. For reasons identified above, ideally such application would occur within the same room as the ensuing treatment to minimize movement; however, if office capacity precludes that option, an alternative is to use another dedicated room for this purpose, during which a thorough cleaning and disinfecting process can be completed of the treatment room between each patient. Following application, patients should be encouraged to continue wearing their masks for the duration of the waiting time until they are ready for the actual procedure.

Other pain management options include topically applied cooling gel or ice packs, which typically have a plastic cover that can serve to retain the virus or other contaminants. Wherever possible, consider disposing of these items entirely after each use. If not, care should be taken when reusing these packs to thoroughly cleanse and disinfect before returning to a common freezer unit, perhaps after being placed within a new, single use plastic bag to be used for storage only. Also, be aware patients may lay these items down during treatment and check-out, which also creates a potential risk for reuse. If disposing of otherwise reusable cold packs is impractical within a particular office, an effective alternative could simply be the single use of double-bagged ice, which in many cases is colder and longer lasting.

Nitrous oxide inhalational analgesia is occasionally used in aesthetic practices and creates an exhaled gas that is directional in nature. For the reasons set forth below, the use of this pain management modality should be reduced to a minimum given the current COVID-19 environment. Patients receiving this analgesic treatment may inhale on a regular basis throughout the procedure. While some clinics deliver this gas mixture using a traditional facial mask (similar to mask oxygen delivery in hospital settings), the most common method of delivery is a disposable plastic mouthpiece. These mouthpieces will become contaminated with the patient's saliva after the first use, and the mouthpiece is then stored with the device, and this process occurs repeatedly during the course of the treatment, after which the entire breathing mouthpiece and hoses are disposed of. Review of existing procedures and protocols for protection from saliva on the mouthpiece should be performed and adapted as needed for COVID-19 risks. Additionally, the patient is typically encouraged to inhale (and thus exhale) deeply for several times at each use of this gas. This policy should be cautiously reviewed in light of COVID-19 risk data on aerosolized droplets resulting from deep breathing, and any necessary use of this pain management modality should be construed as an AGP and subject to the corresponding highest levels of PPE requirements for AGPs recommended throughout this Guidance, including use of an N95 mask and a face shield.

#### Injectables

Dermal fillers, botulinum toxins, and other similar minimally-invasive facial injectables and other injectable procedures are among the most common treatments performed in many aesthetic offices and are likely to be in great demand by patients who have had their regular treatment cycles interrupted by COVID-19 stay-at-home orders.

These procedures usually take only several minutes of actual injection time but may take longer depending on the type of treatment being performed and the number of treatment areas being injected. Despite the short duration of treatment, anatomic location of injections, largely in the face and neck area, combined with the extremely close proximity to the patient's airways necessary for the high-detail work, create exposure risk. Irrespective of prior practice, post-COVID-19 it is important to deploy adequate PPE for these procedures, at a minimum including the use of a three-ply surgical mask, wraparound safety glasses, gown and gloves for the provider administering the injections, as well as all staff in the treatment room. Wherever possible, it is advisable to consider elevating the PPE set-up for these procedures to include the use of an N95 mask and full goggles and face shields. In all cases, intra-procedure discussion by both provider and patient should be kept to an absolute minimum to reduce the risk of airborne transmission through speaking.^26^

As a general practice, vials and syringes should be laid out and prepared prior to patient entrance into the treatment room to minimize exposure time. Proper hand washing and infection control procedures should also be followed when handling vials and syringes, and when applying ice and topical anesthetic agents. Patients should reapply their masks post-procedure.

Injectable procedures below the clavicle, for example such as sclerotherapy and FDA-pending treatments for cellulite reduction, allow some additional distance from the patient's respiratory pathways; however, they still require close physical contact and risk of disease transmission through airborne droplets in shared airspace due to normal breathing and talking, and further exposure may occur through sneezing and coughing. Therefore, this Guidance recommends the same minimum baseline PPE protocol for all injectable procedures irrespective of anatomical region.

#### Non-invasive body contouring

Because they are largely focused on anatomical regions other than the face, the category of cryolipolysis, radiofrequency, electromagnetic and other similar body contouring and body sculpting procedures often do not involve the same face-to-face proximity between provider and patient during treatment. This is also true because certain body contouring procedures require limited in-room contact with the patient once the device has been applied and the procedure has commenced. That said, all such procedures nonetheless require a provider or staff to interact closely with the patient during set-up and application of the device to the selected treatment areas, and during that time the risk of transmission through breathing, talking, coughing and sneezing is omnipresent. Further, some of these body contouring procedures do involve treatment in the neck area to address submental fat. Accordingly, for all body contouring procedures, this Guidance recommends the same minimum PPE level required as a baseline for any form of office treatment, namely a three-ply surgical mask, wraparound eye protection, gown and gloves for all providers and staff in the treatment room. This consistent approach to PPE prioritizes patient and employee safety, minimizes the risk of errors by attempting to parse PPE levels too finely, and fosters maximum patient confidence in the practice. When contouring procedures are performed above the clavicle, consider heightened PPE including an N95 mask, goggles, and possibly a face shield. For body contouring procedures below the clavicle, it is advisable for patients to remain masked throughout the treatment, and particularly when a provider or staff is in the treatment room.

As mentioned above, following commencement of certain of these procedures, the patient is often in a separate room from the provider while the treatment takes place, which may take 15 to 60 minutes depending on the device and the treatment area. In such scenarios, PPE may be removed upon exiting the treatment room and reapplied on reentry; however, in so doing, it is critical to scrupulously observe proper donning and doffing protocols and associated handwashing requirements, in order to avoid cross-contamination.

With some body contouring devices, it is possible to position a disposable pad between the treatment area and device, a practice that should be followed wherever possible. Often a measuring tape is used in conjunction with these procedures for initial patient assessment, and if so, it is advisable to utilize a single-use measuring tape in this regard, when measurement is needed. To the extent support pillows are used during the procedure, consider using disposable pillows or pillows that have a waterproof, plastic or vinyl covering capable of being thoroughly disinfected. Following each procedure, the entire body contouring device, not simply the contact points, should be comprehensively cleaned and disinfected, using approved disinfecting agents, and in conformance with any manufacturer instructions.

#### Energy-based procedures of the face and neck

Depending on the type of device used, setting and depth of treatment, the various laser, light, heat and other similar energy-based procedures of the face and neck performed in a medical aesthetic office are often mechanically disruptive and thus need to be deployed with a high degree of safety protocols. In addition to the inherent risks associated with the fact that they involve extended contact time at close proximity with patient airways, a number of these treatments may be categorized as non-respiratory AGPs based upon emission of airborne debris particles or other contaminants. For example, certain laser and other energy-emitting device procedures may produce a plume of vaporized and ejected tissue that, even when evacuated by suction, has the potential to exit into the treatment room.^25^ Evacuator suction systems should have adequate and regularly monitored two-stage filtration type, and require frequent inspection and replacement of the filters.

Further, it is common for cooling positive air pressure to be used for pain management during a number of laser and other energy-emitting device procedures, often engineered into the operation of the devices themselves. These devices typically have a control for air speed/velocity. Such positive air pressure increases the risk of transmission; therefore, for those procedures where use of cooling air is a function of patient comfort and not required device safety, consider substituting other forms of pain management where possible to achieve adequate pain control with other modalities. Where such pain control is not possible, and/or if cooling air is required for device safety, consider modulating air speed, duration of use, and vector of flow to reduce usage to a minimum level required for safety and/or comfort.

For all these reasons, consider limiting all such AGPs to one or more designated treatment rooms with appropriate air handling, containment and evacuation systems, in order to avoid exposing other treatment rooms or office areas. Review air filter replacement policies and consider accelerated replacement schedules in consultation with device manufacturers. In the event an office has any treatment rooms equipped with negative air pressure capacity, AGPs should be concentrated in these facilities to the maximum extent possible.

Similarly, the maximum available level of PPE should be deployed for all these energy-based procedures of the face. Minimum required PPE for providers and staff either administering, or otherwise in the treatment room for, these AGPs should include an N95 or equivalent mask, wraparound safety glasses or full goggles, gloves and a gown, and if available a surgical cap. Use of a face shield is also strongly advised. Gloves, gowns and caps used in AGPs should be considered single use only, and eye protection should be thoroughly cleaned and disinfected with an approved disinfectant after each use. It is strongly advised that masks should similarly be disposed after each procedure, unless used under a face shield in conjunction with thorough disinfecting protocols. In all cases, despite PPE utilization, intra-procedure discussion by both provider and patient should be kept to an absolute minimum to reduce risk of airborne transmission through speaking. And other than the provider administering the procedure and the patient, nobody else should be in the treatment room, unless a staff member is required to be present, and then only with full PPE consistent with the provider's set-up.

Special consideration should be given to integrating various PPE elements for safe use in practice during performance of these AGPs. While it is true that all procedures involving use of a mask in combination with protective eyewear carry the risk of a gap or slip mid-procedure that creates an exposure to contaminants, such risk is amplified with these AGPs given the possible presence of plumes and positive air pressure, as well as the contingency of laser energy being misdirected and impairing a provider's vision. It is important that employees are not just educated on proper use of PPE, but also practice integrating kits to ensure comfort, fit, coverage, stability and visibility.

Across all energy-based procedures, comprehensive cleaning and disinfection should occur after each treatment, using approved disinfecting agents and pursuant to manufacturer instructions. This should include both the tip of the handpiece and other patient and operator contact points, as well as the entirety of the device and all surfaces in the treatment room that may have been subject to plume or other positive air pressure displacement effect. It is also critical to establish a protocol for appropriate frequency of sterilization procedures for, and inspection and replacement of, all device filters and cartridges. As an additional final step, disinfect the tip of the handpiece again in front of the next patient prior to the next procedure.

As a result of disruption to the skin barrier following all these treatments, skin may be more susceptible to infection. It is advisable to provide patients with a new, clean face mask following all such procedures. Patients should not reuse the mask they wore into the office, if at all possible.

With respect to patient masks, it also bears noting that, to the extent certain laser, light and other similar energy-based procedures are sometimes performed below the clavicle, patients should wear a mask for the entirety of such procedures. Irrespective of the anatomical area of treatment, however, providers and staff should remain at the highest level of PPE protection described above, as these particular procedures remain properly regarded as AGPs, even when focused on the body.

#### Skin treatment procedures

Skin care treatments encompass a wide range of procedures from those that are non-invasive (e.g., medical facials, water-based facials, chemical peels, and non-ablative fractional resurfacing), to those that are moderately invasive (e.g., microneedling) and may result in a nominal amount of localized (pinpoint) bleeding. Given the positioning of such treatments within a busy aesthetics practice and the designation of staff often responsible for administering them, there may be some tendency to default to a lower level of safety vigilance for such procedures; however, any such impulse should be categorically resisted. These treatments are labor- and time- intensive and may require anywhere from 30–60 minutes of time spent in close proximity to the patient, often with staff hands directly in contact with a patient's face.

Accordingly, in addition to consistent use of proper baseline PPE as with any office aesthetic treatment discussed in this Guidance, it is advisable to limit the number and duration of treatments provided per patient visit, provide pain management through modalities other than cooling fans or handheld cooling devices wherever possible, and minimize intra-procedure discussion by both staff and patient. Additionally, preference should be given to utilizing devices with disposable tips, cartridges, blades, and applicators and mixing bowls.

Within the broader category of skin care treatments, some procedures require additional consideration in the current COVID-19 climate. For example, deeper microneedling may produce bloodborne pathogen risk, and certain micro- and hydra-dermabrasion procedures may actually be properly regarded as non-respiratory AGPs due to risk of emission of airborne particles or contaminants as a result of device features such as positive pressure water jets, closed loop vacuum or other vortex type treatments. In such cases, it is advisable to approach this subset of skin care treatments with the same heightened safety protocols as other energy-based procedures of the face, as outlined above. Thus, in addition to complying with device-specific and room-wide infection control and cleaning protocols, consider limiting use of these procedures to a specific treatment room with appropriate air evacuation systems, and enhancing the type of PPE for all staff in the room (e.g., single-use N95 or equivalent mask, single-use gown, gloves and wraparound glasses or goggles, possibly even in conjunction with a face shield). Also, it is prudent to provide patients with a new, clean disposable face mask following all these procedures. In sum, across the various categories of common office aesthetic procedures discussed throughout this Guidance, the key considerations for enhanced COVID-19 vigilance through PPE selection and disinfection protocols are summarized below (Table 3).

### VII. Conclusions

This AesCert Guidance is intended to supplement other advice offered by professional societies and governmental agencies. It has been deliberated and prepared on a multi-disciplinary basis so as to consider many relevant factors involved in operating an aesthetic practice in a COVID-19 environment, as we today understand the virus and its contagious properties.

Progress will be made in the months ahead in testing capability, both for active disease and antibody production. Similarly, progress is likely in clinical evaluation of drug therapies and, ultimately, development of a vaccine. It is incumbent upon every practitioner to stay abreast of these developments as they will affect the practice of aesthetic medicine and patient care and safety in important ways.
